# Progress in Diabetes

**Published:** 1901-07-27

**Authors:** 


### PROGRESS IN DIABETES.

**Acute Infective Rheumatism.**—Drs. Poynton and Paine[1] have continued their researches upon the bacteriology of this disease, and have brought further evidence that the diplococcus which they have isolated is the active cause of, at least, some of the cases of acute rheumatism in this country. They had already demonstrated the presence of this micro-organism in the rheumatic nodule, and now they have succeeded in isolating it from a nodule, and with this culture they have produced a typical attack of rheumatism in a rabbit. A nodule was excised from the elbow of a child that had died two hours before of rheumatic fever. This was placed in a medium of milk and bouillon rendered slightly acid with lactic acid and incubated anaërobically. On the third day a growth of diplococci was found and sub-cultures made on blood-agar.

An intravenous inoculation into a rabbit was then made from these, producing the following results. On the first day rapidity of the heart, and stiffness of the hind limbs on the second. A week later the murmur had ceased, but the left hind and fore limbs were weakened and stiff, and this rapidly spread to all four limbs. After a fortnight from the inoculation there were no joint symptoms, but there was a definite systolic murmur, with signs of pericarditis. A further inoculation was made to revive the active condition, and the animal killed a few days later. There was found endocarditis, pericarditis, and effusion into a joint, and from the latter a pure culture was obtained. In this case, therefore, the authors have fulfilled Koch's postulates.

The organisms have been found in the cortex and meninges of the brain in a rabbit that showed choreic movements after inoculation, and in a résumé of previous bacteriological research by others they mention that Dana isolated a diplococcus in the meninges, and Apert a diplococcus from the brain, the latter author considering this organism to be identical with that of Triboulet in rheumatism. Wassermann and Malkoff also isolated a diplococcus from a case of chorea, and produced rheumatism in rabbits by inoculation with it.

As regards the rôle played by fright in the causation of chorea, Drs. Poynton and Paine state that "when chorea arises in a rheumatic child, and is attributed to fright, it would seem probable that fright acts upon the rheumatic brain much as strain acts upon the rheumatic heart—namely, intensifies the rheumatic process in the brain and precipitates chorea, as does strain hasten the onset of rheumatic carditis." They have also been able to produce in a rabbit malignant endocarditis with this organism, and then a simple endocarditis with the same, indicating a variability in virulence. In two cases ante-mortem clot was formed in the heart, and in one of these cases the animal was killed to verify their diagnosis of this condition. The symptoms upon which the diagnosis was made were:—"Rapid, feeble, distant heart-sounds with the development of a systolic murmur. . . " In a paper read at the debate on rheumatism at the Chelsea Clinical Society,[2] the same authors state that the white infarcts found in the kidneys are due to an invasion of diplococci, and not to a gross embolism, the cloudy swelling passing on to necrosis. They have demonstrated the diplococcus in the urine of a case of rheumatic pericarditis, and more recently in the kidney and urine after death.

Dr. T. K. Monro[3] records a case of atrophy of the testicle resulting from orchitis due to rheumatism. The patient complained of pain in the testicle on the eleventh day of the attack of rheumatism, and on the next day there was swelling and tenderness. In a few days the pain ceased, and the swelling gradually subsided and was normal in little over a month. Two months later the testicle was found to be one-third the size of the unaffected one. In a review of orchitis occurring in rheumatism it is found to be a very uncommon complication of rheumatism, the author finding references to it by Robert Macleod, Besnier, and W. J. Penny of Bristol.

Mircoli,[4] looking upon acute rheumatism as being of the nature of a pyæmia, has made experiments to find how the various organs of the body differ as media for the growth of organisms. The organism that he selected was a staphylococcus. This was injected intravenously into rabbits, and later the animals were killed and the organs incubated without any further additions. The rapidity of growth was found to be in the following order of luxuriance—kidney, heart, liver, spleen, blood—but in virulence of culture blood came before liver and spleen. He considers that some cases of obscure kidney disease are a consequence of this marked superiority of the kidney as a nutrient medium for the infective agent of rheumatism. In the treatment of acute rheumatism D. T. Kyner[5] recommends the use of asperin, "acetyl salicylic acid," in place of salicylates on the ground that the former has an agreeable taste, does not irritate the stomach, does not cause tinnitus aurium "after the administration of physiological doses"; does not cause cardiac depression. It does not impair the appetite even during prolonged use. Haig,[6] holding that rheumatism is gout and gout rheumatism, considers that salicylates given with alkalies is useless in rheumatism, the latter, although themselves solvents of uric acid, destroying the solvent action of salicylates on uric acid. Hector Sarafis[7] recommends the use of petroleum applied locally with massage for ten minutes once or twice a day. He considers it to be sufficient to cure without resorting to any internal treatment; it lowers the temperature in a short time and is cheap. He explains its action as being due to its antiseptic properties, and considers that it has an action upon the nerves of the synovial membranes.

**Gout and Plumbism.**—Dr. George Lorimer[8] has made an inquiry as to how far plumbism affects the course of gout when these two are combined, and whether there are any symptoms which especially distinguish this combination. He makes an analysis of 107 cases of gout in which there was also lead impregnation, including in this list 27 cases where there was plumbic arthritis rather than true gout, believing these to be gouty rather than rheumatic from having observed cases that at first could not be distinguished from rheumatism gradually change into typical cases of gout. As regards the age of the patient, the average onset was early compared with that of gout, 70 cases having the first attack before 35 years of age, whereas in gout the occurrence before that age is unusual without a strong hereditary predisposition. The hereditary tendency was noted as present in only 9 cases as compared with 50 per cent. in gout. Anæmia was present in 75 cases, contrasting strongly with ordinary gout. The type of arthritis in the maj ority of cases was asthenic, pyrexia slight, and the local and constitutional phenomena less intense, but more lingering and obstinate. In only one case was there eczema, contrasting markedly with ordinary gout with 18 per cent. Arterio-capillary fibrosis along with artheroma was noted in two-thirds of the cases as strongly marked, and present to a lesser degree in most of the others. Albuminuria occurred in 83 per cent. of these cases, as compared with 26 per cent. in chronic gout. In 28 cases there was chronic renal disease, and of these it occurred before the age of 30 in 13 cases. The author believes that the arthritic diathesis is an essential factor.

[1] Lancet, May 4, 1901. [2] Clin. Jour., May 8, 1901. [3] Glasgow Med. Jour., June, 1901. [4] Brit. Med. Jour., Feb. 9, 1901. [5] South Illinois Jour. Med. and Surg.. March, 1901. [6] Med. Rec., Jan. 26, 1901. [7] Amer. Jour. Med. Sci., May, 1901. [8] Quar. Med. Jour., May, 1901.

